# Giant Postischemic Left Ventricular Aneurysm Without Q Waves: Multimodality Imaging-Guided Surgery

**DOI:** 10.1016/j.jaccas.2026.108319

**Published:** 2026-05-14

**Authors:** Luca Gaido, Matteo Attisani, Fabrizio Scalini, Alberto Pacielli, Laura Ravera, Chiara Calcagnile, Alessandra Rubino, Massimo Giammaria

**Affiliations:** a Division of Cardiology, Martini Hospital, Turin, Italy; b Division of Cardiac Surgery, San Giovanni Bosco Hospital, Turin, Italy; c Department of Radiology and Neuroradiology, San Giovanni Bosco Hospital, Turin, Italy; d Division of Cardiology, Maria Vittoria Hospital, Turin, Italy; e Division of Cardiology, San Giovanni Bosco Hospital, Turin, Italy

**Keywords:** cardiac magnetic resonance, computed tomography, echocardiography, electrocardiogram, left ventricle, myocardial infarction, percutaneous coronary intervention, thrombus, ultrasound

## Abstract

**Background:**

Left ventricular aneurysm (LVA) is a mechanical complication of myocardial infarction, typically associated with anterior infarction and diagnostic electrocardiographic changes. However, nonanterior aneurysms may present with misleading findings, delaying diagnosis.

**Case Summary:**

A 55-year-old woman with inferolateral myocardial infarction underwent successful percutaneous coronary intervention. Despite enzymatic evidence of necrosis, serial electrocardiograms showed no pathological Q waves. Multimodality imaging revealed progressive left ventricular remodeling with aneurysmal evolution and mural thrombus formation. Echocardiography documented transition from regional dysfunction to aneurysm, while cardiac magnetic resonance confirmed transmural fibrosis and excluded viability and pseudoaneurysm. Surgery was initially deferred but was later expedited owing to thrombus progression. Ventricular reconstruction with aneurysm exclusion and mitral annuloplasty was successfully performed.

**Conclusions:**

LVAs may develop without pathological Q waves. Multimodality imaging is essential for diagnosis, tissue characterization, and surgical decision-making.

**Take-Home Messages:**

LVA may develop without pathological Q waves, particularly in nonanterior myocardial infarctions, potentially delaying diagnosis. Serial multimodality imaging is essential to identify aneurysm progression, detect mural thrombosis, and guide individualized timing of surgical intervention.

## Visual Summary

**Figure:**
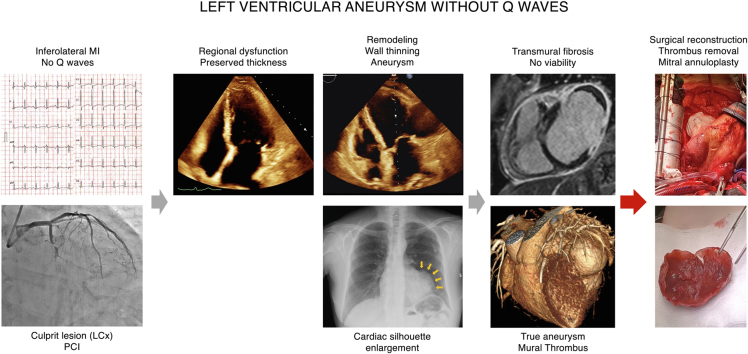

Left ventricular aneurysm (LVA) is a recognized mechanical complication of myocardial infarction, typically associated with anterior infarction and characteristic electrocardiographic changes. However, aneurysms involving nonanterior segments may present with atypical or misleading findings, potentially delaying diagnosis and management. This case highlights the role of serial multimodality imaging in detecting progressive ventricular remodeling and guiding timely surgical intervention in an unusual presentation without pathological Q waves.

**Take-Home Messages**

• Left ventricular aneurysm may develop without pathological Q waves, particularly in nonanterior myocardial infarctions, potentially delaying diagnosis.
• Serial multimodality imaging is essential to identify aneurysm progression, detect mural thrombosis, and guide individualized timing of surgical intervention.

## Case Presentation

A 55-year-old woman presented to the emergency department with mild dyspnea and fatigue, in the setting of a recent febrile episode. Her medical history was notable for dementia with Lewy bodies. Although the patient was awake and cooperative, symptom reporting was limited, resulting in increased complexity of history-taking. Laboratory testing revealed elevated high-sensitivity troponin T (1,525 ng/L) along with increased inflammatory markers, including C-reactive protein (13 mg/L), while other routine laboratory parameters were unremarkable. Surface electrocardiogram showed flat and negative T waves in the inferolateral leads, without pathological Q waves (Figure 1).

**Figure 1:**
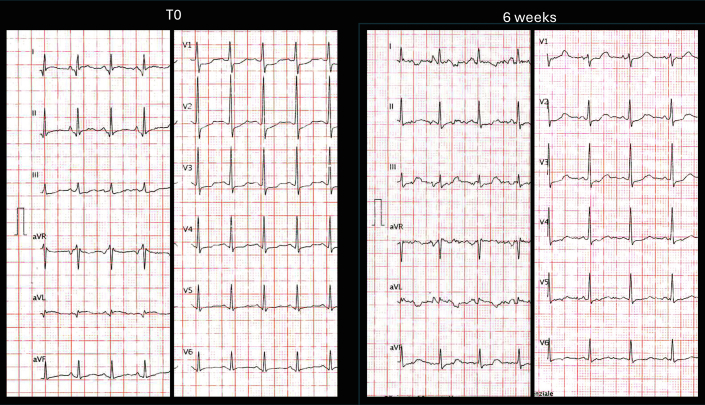
Electrocardiographic Evolution Baseline (T_0_) electrocardiogram and follow-up tracing at 6 weeks showing sinus rhythm with nonspecific ST-T abnormalities in inferolateral leads and absence of pathological Q waves, despite subsequent development of a giant left ventricular aneurysm.

Coronary angiography demonstrated a subocclusive lesion of the proximal obtuse marginal branch of the left circumflex artery, which was treated with drug-eluting stent implantation. A critical stenosis of the left anterior descending artery and diagonal branch was subsequently treated during a staged in-hospital procedure, achieving complete revascularization (Figure 2, Video 1).

**Visual Summary:**
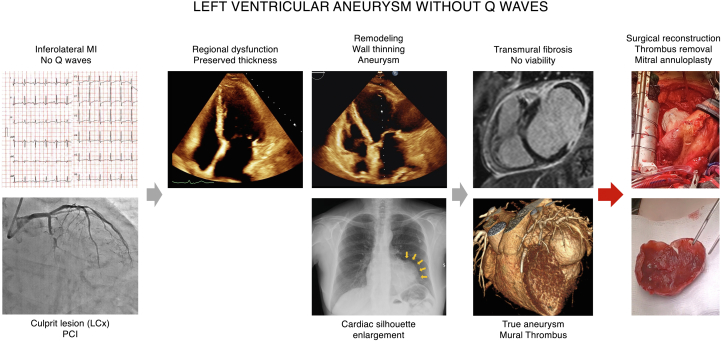
Multimodality Imaging Timeline of Left Ventricular Aneurysm Without Q Waves Schematic timeline illustrating the evolution from inferolateral myocardial infarction without pathological Q waves, despite a culprit lesion in the left circumflex artery treated with percutaneous coronary intervention, to progressive left ventricular remodeling with aneurysm formation. Serial multimodality imaging demonstrates transition from regional dysfunction with preserved wall thickness to wall thinning and aneurysmal deformation, followed by transmural fibrosis without viability on cardiac magnetic resonance and development of a true aneurysm with mural thrombus. Progressive cardiac enlargement is observed on chest radiography. Surgical ventricular reconstruction with thrombus removal and mitral annuloplasty is subsequently performed. LCx = left circumflex; MI = myocardial infarction; PCI = percutaneous coronary intervention.

**Figure 2:**
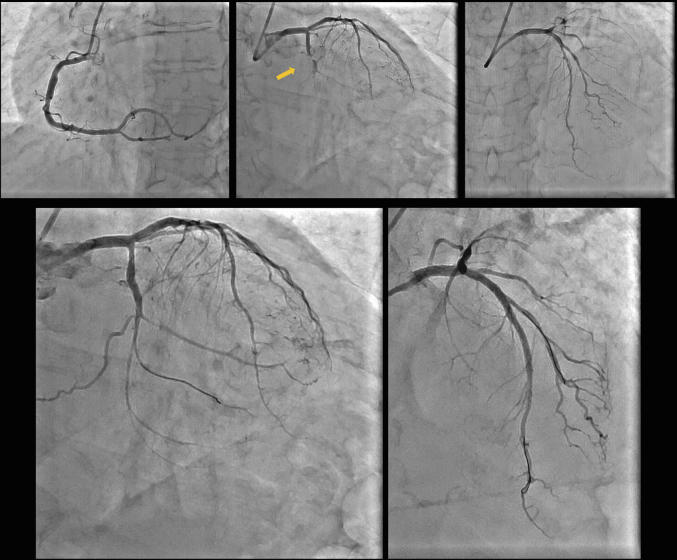
Coronary Angiography and Revascularization Coronary angiography during the acute phase showing multivessel coronary artery disease with a subocclusive lesion of the obtuse marginal branch of the circumflex artery (culprit lesion, arrow), treated with percutaneous coronary intervention. (Bottom) Final panels demonstrate staged in-hospital complete revascularization of the left anterior descending artery and diagonal branch.

Early transthoracic echocardiography revealed reduced left ventricular systolic function (ejection fraction: 43%) with akinesia of the midbasal anterior, anterolateral, and inferolateral segments, while myocardial wall thickness remained preserved. Serial imaging at 1, 4, 5, and 7 weeks after the index myocardial infarction enabled precise characterization of disease progression from initial regional dysfunction to progressive ventricular remodeling, aneurysm formation, and mural thrombus development. Subsequent echocardiographic examinations demonstrated a stepwise evolution toward aneurysmal deformation, with progressive wall thinning, outward bulging, and adverse remodeling (Figure 3, Video 2). These findings initially raised suspicion of myocardial stunning or hibernation, which was subsequently excluded on the basis of multimodality imaging.

**Figure 3:**
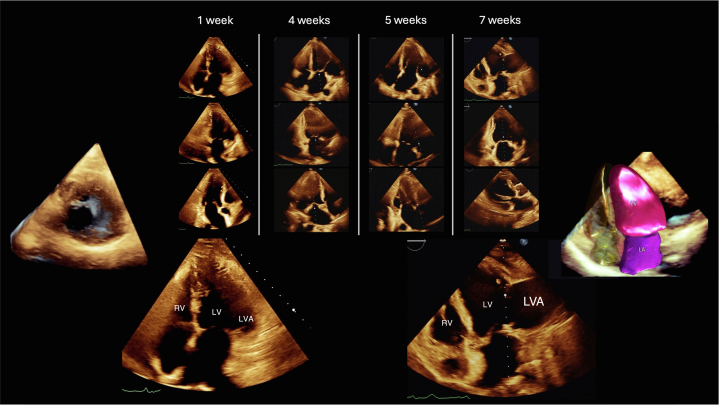
Serial Transthoracic Echocardiography Showing Progressive Left Ventricular Remodeling and Aneurysm Formation Sequential apical views obtained at 1, 4, 5, and 7 weeks after the index myocardial infarction demonstrate the temporal evolution from initial regional wall motion abnormalities with preserved wall thickness in the basal inferolateral and anterolateral segments to progressive wall thinning, outward bulging, and formation of a large left ventricular aneurysm. Lower panels highlight the aneurysmal cavity (LVA) with associated chamber deformation. LV = left ventricle; LVA = left ventricular aneurysm; RV = right ventricle.

Concomitantly, serial chest radiographs showed progressive distortion of the left cardiac silhouette, consistent with structural remodeling (Figure 4).

**Figure 4:**
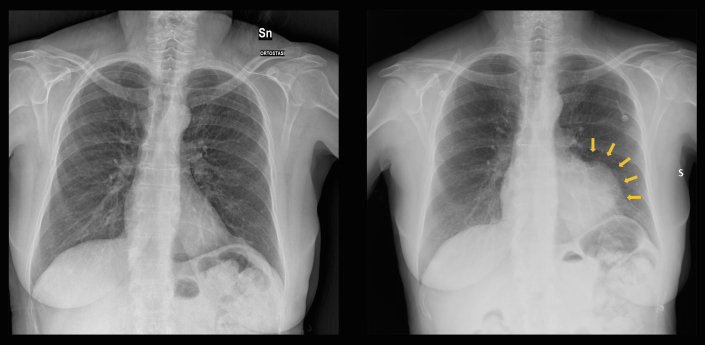
Chest X-Ray Evolution Chest radiographs obtained (Left) early after myocardial infarction and (Right) at follow-up showing progressive distortion of the left cardiac silhouette, consistent with macroscopic evolution of a left ventricular aneurysm (arrows).

Cardiac computed tomography (CT) was performed to further define ventricular anatomy. The first examination demonstrated a giant LVA with a wide basal neck, predominantly involving the basal inferolateral and basal anterolateral walls, with extension to the mid inferolateral segment. A subsequent preoperative CT documented interval development of mural thrombus within the aneurysmal cavity (Figures 5A and 5B).

**Figure 5:**
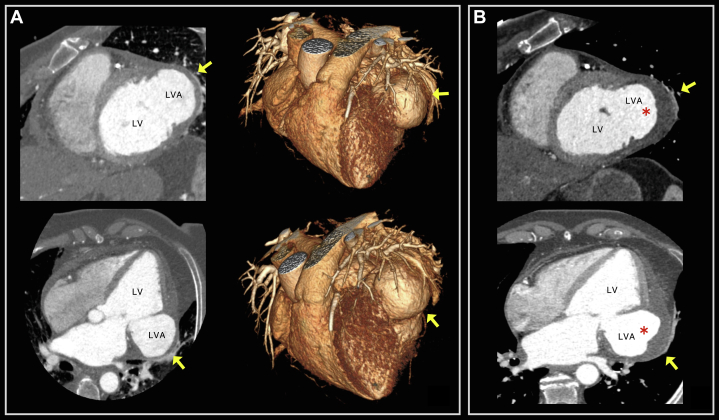
Cardiac Computed Tomography Evaluation of Left Ventricular Aneurysm Progression (A) Initial contrast-enhanced cardiac computed tomography (left panels) showing a giant true left ventricular aneurysm with a wide basal neck involving the inferolateral and anterolateral walls, associated with marked wall thinning and outward bulging (arrows), without evidence of mural thrombosis. Three-dimensional volume-rendered images (right panels) further delineate aneurysm morphology. (B) Preoperative follow-up cardiac computed tomography demonstrating interval development of an extensive laminated mural thrombus adherent to the aneurysmal cavity (asterisk), confirming progressive aneurysmal remodeling. Arrows indicate the aneurysmal segment. LV = left ventricle; LVA = left ventricular aneurysm.

Cardiac magnetic resonance (CMR) confirmed severe wall thinning and dyskinetic motion of the aneurysmal segment. Late gadolinium enhancement demonstrated transmural fibrotic replacement of the aneurysmal wall and revealed an extensive adherent mural thrombus. T2-weighted imaging showed no myocardial edema, excluding myocardial stunning or hibernation (Figure 6, Video 3).

**Figure 6:**
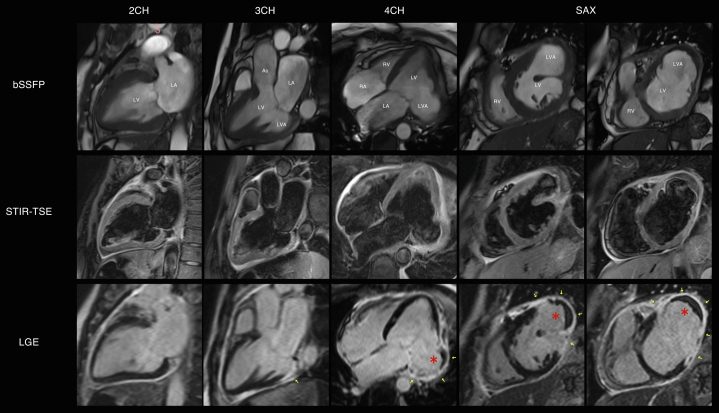
Cardiac Magnetic Resonance Imaging Characterization of Left Ventricular Aneurysm and Mural Thrombus (Top Row) Balanced steady-state free precession (bSSFP) cine images demonstrate a large inferolateral left ventricular aneurysm with associated chamber remodeling. (Middle Row) Short-tau inversion recovery–turbo spin echo (STIR-TSE) sequences show no significant myocardial edema. (Bottom Row) Late gadolinium enhancement (LGE) reveals transmural fibrosis involving the basal inferolateral and basal anterolateral walls, with extension to the mid-inferolateral segment (arrows), and a large adherent mural thrombus (asterisk). 2CH = 2-chamber view; 3CH = 3-chamber view; 4CH = 4-chamber view; Ao = aorta; LA = left atrium; LVA = left ventricular aneurysm; LV = left ventricle; RA = right atrium; RV = right ventricle; SAX = short-axis view.

Surgical intervention was initially deferred to allow tissue stabilization during the subacute phase. Following the identification of a left ventricular mural thrombus on subsequent imaging, anticoagulation therapy was initiated to mitigate thromboembolic risk. Multimodality imaging provided incremental characterization of the aneurysmal cavity, with clear identification of a large adherent thrombus within a complex inferolateral aneurysm. In this anatomical context, the extent of thrombotic burden was considered to confer a high embolic risk, despite the absence of formal quantitative risk-stratification tools. Surgery was ultimately performed approximately 2 months after the index myocardial infarction, after completion of the subacute healing phase. Based on the integrated clinical and imaging findings, the heart team decided to proceed with surgical intervention, as the presence of a large aneurysm and substantial thrombotic burden was considered to outweigh the risks of continued conservative management.

The procedure was performed via median sternotomy under cardiopulmonary bypass. After left ventriculotomy, a giant true aneurysm involving the basal inferolateral, basal anterolateral, and mid inferolateral segments was identified. The aneurysmal wall appeared markedly thinned and fibrotic, and an extensive laminated mural thrombus firmly adherent to the aneurysmal cavity was completely removed (Figure 7, Video 4). A wide basal neck confirmed the diagnosis of true aneurysm.

**Figure 7:**
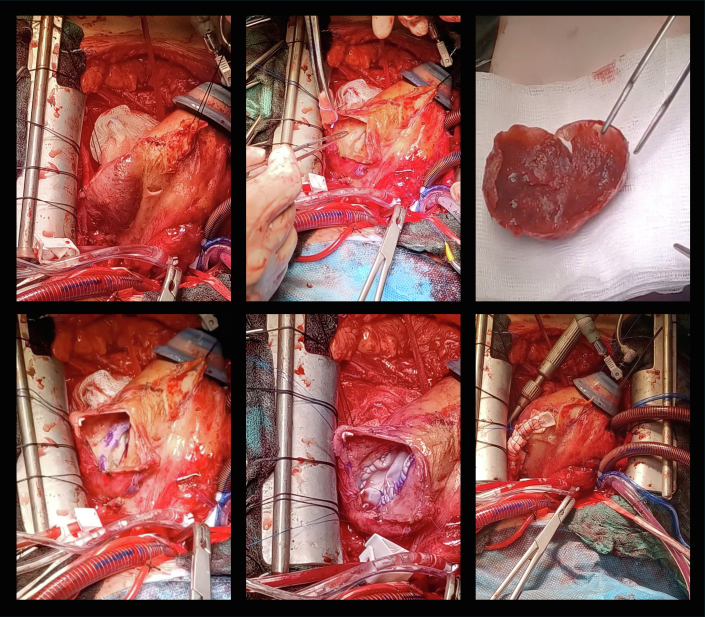
Intraoperative Findings Intraoperative photographs confirming the presence of a giant true left ventricular aneurysm with markedly thinned and fibrotic walls. Surgical opening of the aneurysm cavity reveals an extensive adherent mural thrombus, followed by aneurysm resection and ventricular reconstruction.

Left ventricular reconstruction was achieved by aneurysm exclusion using a heterologous pericardial patch sutured to the fibrotic margins of the aneurysm neck and reinforced with surgical adhesive. The ventricular wall was closed with double-layer reinforced sutures to restore ventricular geometry. Concomitant mitral valve annuloplasty was performed using a posterior annuloplasty ring to correct functional mitral regurgitation secondary to papillary muscle displacement. Intraoperative transesophageal echocardiography confirmed effective aneurysm exclusion and satisfactory mitral valve competence.

## Discussion

This case illustrates the evolution of postischemic left ventricular remodeling and highlights key diagnostic and therapeutic considerations.

First, giant postischemic LVAs may develop without pathological Q waves, particularly when involving nonanterior territories such as the inferolateral wall. In these settings, surface electrocardiography may markedly underestimate the extent of structural myocardial damage.^1^, ^2^, ^3^ The absence of Q waves despite transmural fibrosis likely reflects the vectorial orientation of electrical forces in nonanterior infarcts, as well as heterogeneous scar formation with partial preservation of viable myocardium, resulting in incomplete electrical silencing. Progressive remodeling may further contribute to a dissociation between structural damage and its electrocardiographic expression. In the present case, serial electrocardiograms showed only nonspecific ST-T abnormalities in the lateral leads (I and aVL), without development of Q waves, despite extensive transmural fibrosis, aneurysm formation, and mural thrombus. This marked discrepancy underscores the limitations of electrocardiography in selected anatomical settings and highlights the need for multimodality imaging for accurate assessment.

Second, early differentiation between reversible ischemic dysfunction and irreversible remodeling may be challenging. Initial echocardiographic findings with preserved wall thickness can suggest myocardial stunning or hibernation. In this case however, serial imaging rapidly demonstrated progressive wall thinning and aneurysmal deformation, features incompatible with reversible ischemic injury. Completion of coronary revascularization was performed as part of a staged strategy rather than in response to suspected viability, as the evolving aneurysmal territory consistently corresponded to the circumflex distribution.^4^

Multimodality imaging played a pivotal role. Echocardiography provided dynamic temporal assessment, chest radiography revealed early macroscopic remodeling, cardiac CT precisely defined aneurysm geometry and documented thrombus progression, and CMR conclusively characterized tissue composition and thrombotic burden.^1^, ^2^, ^3^^,^^5^, ^6^, ^7^

True LVA and pseudoaneurysm represent distinct pathological entities that can be differentiated using multimodality imaging. On echocardiography, true aneurysms typically present with a wide neck and dyskinetic but continuous myocardial wall, whereas pseudoaneurysms are characterized by a narrow neck and abrupt discontinuity of the myocardial wall with containment by pericardial structures. Cardiac CT provides detailed anatomical assessment, allowing precise evaluation of aneurysm geometry, neck size, and relationship with surrounding structures, as well as identification of mural thrombus. CMR plays a pivotal role in tissue characterization: True aneurysms demonstrate transmural late gadolinium enhancement consistent with fibrotic myocardial replacement, while pseudoaneurysms lack myocardial tissue in the aneurysmal wall and are typically contained by pericardium. In the present case, the presence of a wide neck, preserved myocardial continuity, absence of pericardial containment, and transmural fibrosis allowed confident diagnosis of a true aneurysm.^1^, ^2^, ^3^^,^^5^

Surgical ventricular reconstruction remains an established therapeutic option in selected patients with postinfarction aneurysm and adverse remodeling.^8^, ^9^, ^10^, ^11^, ^12^ The timing of intervention must be individualized. In clinically stable patients, surgery is often performed after completion of the subacute healing phase, when infarcted myocardium has undergone fibrotic stabilization, facilitating secure reconstruction. However, early surgery may be appropriate in selected cases presenting with progressive heart failure, ventricular arrhythmias, or evolving intraventricular thrombus. Contemporary series have demonstrated that ventricular aneurysm repair within 30 days after acute myocardial infarction can be feasible with acceptable outcomes in carefully selected patients.^13^

In the present case, surgery was initially deferred. However, documented progression of mural thrombus prompted expedited intervention, balancing technical considerations with embolic risk.

## Conclusions

Giant postischemic LVA may evolve rapidly and silently, even in the absence of classical electrocardiographic markers of necrosis. Multimodality imaging, integrated with surgical assessment, is critical for accurate diagnosis, differentiation from reversible dysfunction and pseudoaneurysm, and for guiding individualized management strategies.

## Funding Support and Author Disclosures

The authors have reported that they have no relationships relevant to the contents of this paper to disclose.

## Appendix

Video 1 Coronary Angiography Coronary angiography demonstrating the proximal subocclusive lesion of the left circumflex artery identified as the culprit lesion, followed by angiographic result after percutaneous coronary intervention.

Video 2 Transthoracic Echocardiography Transthoracic echocardiography showing the left ventricular aneurysm involving the midbasal inferolateral and anterolateral segments, with wide neck, wall thinning, and dyskinetic motion, consistent with true aneurysm formation.

Video 3 Cardiac Magnetic Resonance Cardiac magnetic resonance cine and late gadolinium enhancement sequences demonstrating transmural scar of the aneurysmal wall and a large adherent mural thrombus, excluding pseudoaneurysm and myocardial viability in the affected segments.

Video 4 Surgical Procedure Intraoperative video documenting surgical exposure and resection of the left ventricular aneurysm, with direct visualization of the fibrotic aneurysmal wall and organized mural thrombus.
